# Gastric Cancer Mortality Trends in the Southern Cone: Disentangling age, period and cohort patterns in Argentina and Chile

**DOI:** 10.1038/s41598-020-58539-w

**Published:** 2020-01-30

**Authors:** Maria del Pilar Diaz, Gloria Icaza, Loreto Nuñez, Sonia A. Pou

**Affiliations:** 1 Instituto de Investigaciones en Ciencias de la Salud (INICSA), Universidad Nacional de Córdoba, Consejo Nacional de Investigaciones Científicas y Técnicas (CONICET), Facultad de Ciencias Médicas; Estadística y Bioestadística, Escuela de Nutrición, Facultad de Ciencias Médicas, Universidad Nacional de Córdoba. Bv. de la Reforma, Ciudad Universitaria, CP 5016 Córdoba, Argentina; 2 Instituto de Matemática y Física, Universidad de Talca, Talca, Chile; 3 Programa de Investigación Asociativa en Cáncer Gástrico, Avenida Lircay s/n, Talca, Chile; 4 Departamento de Salud Pública, Facultad de Ciencias de la Salud, Universidad de Talca, Talca, Chile

**Keywords:** Gastric cancer, Preventive medicine, Epidemiology, Epidemiology, Cancer epidemiology

## Abstract

Although Argentina and Chile are neighboring countries, gastric cancer (GC) is the first cancer death cause in the Chilean male population, while it is ranked in fifth place for Argentinean males. This study is the first to identify the differential time-patterns associated with the age-period-cohort effects for the last few decades (1990–2015) in these Southern Cone countries. Trends of age-standardized truncated mortality rates (ASMR) for GC were analyzed using log-linear Poisson age-period-cohort models, including cubic splines for each component. The ASMR trends for GC decreased in both sexes but more considerably in Chile and more favorably for males (annual percentage changes 2002–2015 = −3.5, 95%CI: −3.9 to −3.1). Moreover, GC age-specific mortality rates were noticeably higher in Chile. A favorable decreasing mortality risk throughout the periods (from 2000) and by cohort was observed for both countries; however, the risk reduction has stabilized in younger female cohorts since 1950-cohort. In conclusion, overall favorable decreasing trends for GC mortality were found; however, when age-period-cohort effects were disentangled, Chile and younger female cohorts showed a more unfavorable scenario. Obesity, lifestyles, and environmental conditions (like altitude) may explain country differences. This analytical approach may be a valuable tool to be replicated in other countries with no population-based cancer registries and acceptable mortality data quality.

## Introduction

Gastric cancer (GC) is one of the five most frequent cancers (excluding non-melanoma skin cancer) in the world^1,2^ and the third leading cause of cancer death in both sexes worldwide^1^. Although GC mortality rates have been declining over the last few decades, the rates in several countries in South America and Asia remain the highest in the world^3^.

The number of deaths in Latin America and the Caribbean attributable to this cancer is among the highest in the world; South America is the third region with one of the highest age-standardized (World Standard Population) incidence rates for GC, preceded only by Eastern Asia and Central and Eastern Europe^1^. Characteristics of the Southern Cone that distinguish it from the northern area of South America include unusually high consumption of meat, mainly barbecued, and wine consumption, which has already been reported as associated with cancer^4^. However, Chile and Argentina are among the countries in South America with the highest and lowest age-standardized mortality rates (ASMR) for both sexes, respectively (ranked in third and 23th place among the 32 countries in the region), with overall ASMR equals of 17.9 and 6.4 per 100,000 in men and women for Chile, and 7.5 and 3.0 for Argentina, respectively^5^. Thus, even though they are neighboring countries in the Southern Cone with similar reports of the human development index and behaviors, the health differences between these countries should be analyzed. *Helicobacter pylori* (*H. pylori*) infection is a primary risk factor of GC. It is estimated that about half of the world’s population is infected with this bacterium^6^, although GC occurrence cannot be explained by *H. pylori* infection alone. Damage to the mucosal barrier due to *H. pylori*-induced inflammation enhances the carcinogenic effect of other risk factors^7^. Substantial epidemiological evidence strongly suggests that GC risk increases with high salt intakes and decreases with high intakes of fruit and vegetables^8^. Changes in alcohol consumption and tobacco smoking may also play a role^8^.

To date, several studies have analyzed GC mortality trends, splitting the temporal variations into the recognized time-components, such as age, cohort of birth, and period of death. Cohort effects may provide some evidence of early-stage carcinogens or risk factors that may influence GC mortality, and period effects may demonstrate the effects of screening and adjuvant treatment on GC mortality. No systematic epidemiological study has been conducted that describes recent and temporal trends in GC mortality in South American countries. In this paper, we explore GC mortality trend differences between Chile and Argentina in the 1990–2015 period, identifying the time patterns associated with age, period, and birth cohort effects.

## Methods

Mortality data was drawn from death certificates provided by the Ministry of Health for Chile and the National Health Ministry for Argentina, for the study period (1990–2015 for Chile and 1991–2015 for Argentina). GC deaths were classified by C16 (ICD-10) and 151 (ICD-9) codes.

Chilean population data from the 1990–2012 period was obtained from the 1992 and 2002 Census intercensal estimations, and from the 2013–2015 period from administrative data provided by the National Institute of Statistics (INE) and the Department of Statistics and Health Information (DEIS). Exponential interpolation from 1991, 2001, and 2010 population census data reported by the National Statistics and Census Institute (INDEC) was used for data for Argentina. Truncated (30–79 years old) age-standardized (World Standard Population) mortality rates (ASMR, per 100,000 person-years) from GC were estimated by sex for all years for each country.

The datasets analyzed during the current study are available from the corresponding author on request.

### Compliance with ethical standards

The present study follows the ethical standards of the institutional research committee and the 1964 Declaration of Helsinki and its later amendments. No ethical approval was required by our institutions as the research involved anonymized records and datasets available in the public domain. Specifically, the mortality databases used in this study do not contain any information that may identify the research subjects, and the data is available from the Health Statistics and Information Division for Argentina and Chile (called DEIS) upon reasonable request and with permission from the Ministries of Health from these countries (publicly available online at www.minsal.cl for Chile). Formal consent is not required for this type of study.

### Statistical analysis

Joinpoint regression analysis^9^ was performed to achieve greater descriptive accuracy of overall temporal trends. Models were fitted to ASMR by sex and separately for each country, providing estimates of the annual percent change (AnPC) and its 95% Confidence Interval (95%CI). Joinpoint regression program, Version 4.6.0.0. (Statistical Research and Applications Branch, National Cancer Institute; 2018) was used.

By considering age, calendar period, and birth cohort, Age-Period-Cohort (APC) models incorporated historical or continuous changes in these components. These models suffer from an identifiability problem (cohort = period–age); therefore, different approaches for fitting were suggested. We chose the Carstensen proposal^10^, which deals with age, period, and cohort as continuous variables (not as “class variables” or factor) and uses appropriated spline functions to represent them. Thus, the general form of a multiplicative age-period model for rates, $\log \,[\lambda (a,p)]=f(a)+g(p)+h(c),$ where $f(a),\,g(p),\,h(c)$ are restricted cubic (natural) splines functions for age, period, and cohort terms within a GLM (Generalized Linear Models) framework with a Poisson family error structure, a log link function, and an offset of log(person risk-time) as exposure were used. The model was adjusted for sex covariate, and country effect for the complete mortality dataset was also included. Fitting the country effect in a single model allowed the calculation of the time-dependent mortality rate ratio (MRR) for the countries by age. After fitting, the age effects were calculated as rates for the reference period in the age–period model, while the period (or cohort) effects were calculated as rate ratios (RR) relative to the reference period (or cohort). The best-fitting model was defined according to the Akaike information criterion (AIC). Rutherford Stata code^11^ was used for fitting all models in Stata 14.0.

## Results

### Joinpoint regression by country

Between 1990 and 2015, a total of 54,857 deaths from GC were registered in Chile and 54,540 in Argentina between 1991 and 2015. About 70% of these deaths were male and 30% female, in both countries. These percentages of deaths by sex were also observed in the first and last years of the study period. GC mortality rates in Chile, as well as in Argentina, revealed higher values for men than women between 30 and 79 years old (3:1 relationship in1990 and 2015), and the lowest rate was seen in 2015 (versus 1990, in both countries and sexes) (Table 1).

**Table 1:** Tendency on age-standardized (World Standard Population) death rates per 100, 000 person from gastric cancer (truncated 30–79 years), between 1990 and 2015 in Argentina and Chile.

Country	Sex	Age-standardized mortality rate		Tendency	
		1990	2015	Years	AnPC (95% CI)
Argentina	Female	8.56	5.98	1991–2003 2003–2015	−2.1 (−2.6; −1.6)^a^ −0.8 (−1.3; −0.3) ^a^
	Male	24.77	15.48	1991–2015	−2.0 (−2.2; −1.9)^a^
Chile	Female	11.04	5.47	1990–2015	−2.6 (−2.8; −2.4)^a^
	Male	28.48	14.96	1990–2002 2002–2015	−1.7 (−2.2; −1.2)^a^ −3.5 (−3.9; −3.1)^a^

AnPC, annual percent change; CI, confidence interval.

^a^Estimated AnPC is significantly different from zero at the alpha 0.05 level.

The ASMR of men and women for GC of both countries showed a decreasing trend. The main decrease was observed in males in Chile (from 2002, −3.5 AnPC; 95%CI: −3.9 to −3.1; p < 0.001). Nonetheless, a significant negative slope was also observed for Chilean females (1990–2015, −2.6 AnPC; 95%CI: −2.8 to −2.4; p < 0.001). In Argentina, the main descending slope was found in females for the 1990–2003 period (−2.1 AnPC; 95%CI: −2.6 to −1.6; p < 0.001). Likewise, a negative slope, with no breakpoint, was found in males (−2.0 AnPC; 95% CI: −2.2 to −1.9; p < 0.001) for the whole analysis period (Table 1).

Any tabulation of mortality data represents an information loss; thus, we decided to show period-cohort Crude Mortality Rates (CMR) to give an overview of rates and trends. Overall, the magnitudes of CMR were lower and more homogeneous for Argentina (Tables 2 and 3), over twice as high in males than in females mainly for Chile and for those belonging to birth cohorts up to 1933 (rates ranged from 102 to 303 in 100,000 males compared to 31.3 to 118 in 100,000 females). For both countries, younger male and female birth cohorts showed lower rates even when increasing in age of death (column trend). Nearly parallel patterns followed each age group (diagonal trends) relative to the periods, and periods showed non-linear tendencies through dates of birth (row trends).

**Table 2:** Specific mortality rates per 100,000 person-years of gastric cancer for period-cohort groups by sex in Chile during 1990–2015.

Males	Cohort														
Period	1913	1918	1923	1928	1933	1938	1943	1948	1953	1958	1963	1968	1973	1978	1983
1991–1995	247.0	310.0	236.0	170.0	102.0	53.0	26.5	13.7	5.15	3.2	1.9				
1996–2000		220.0	303.0	226.0	152.0	83.8	50.1	25.1	12.1	5.6	2.3	2.0			
2001–2005			202.0	287.0	209.0	126.0	72.8	39.6	21.0	10.7	5.2	2.5	1.6		
2006–2010				200.0	264.0	172.0	110.0	62.4	32.7	17.4	10.4	4.7	2.0	0.7	
2011–2015					188.0	205.0	139.0	84.0	48.5	28.0	14.0	6.9	3.2	1.7	1.1
Females	Cohort														
Period	1913	1918	1923	1928	1933	1938	1943	1948	1953	1958	1963	1968	1973	1978	1983
1991–1995	99.2	118.0	83.3	54.3	31.3	18.9	9.3	5.8	3.2	2.2	1.3				
1996–2000		71.8	111.0	80.1	45.8	25.7	14.0	8.4	4.4	3.2	1.5	1.7			
2001–2005			72.9	101.0	67.4	38.3	21.7	13.3	7.7	5.3	3.1	1.9	1.8		
2006–2010				72.3	83.8	53.9	34.1	20.0	11.2	7.1	4.4	2.7	1.7	1.2	
2011–2015					60.6	74.9	44.7	30.2	16.2	9.7	6.1	4.8	2.9	1.5	1.2

**Table 3:** Specific mortality rates per 100,000 person-years of gastric cancer for period-cohort groups by sex in Argentina during 1990–2015.

Males	Cohort														
Period	1913	1918	1923	1928	1933	1938	1943	1948	1953	1958	1963	1968	1973	1978	1983
1991–1995	108.0	100.0	75.2	53.9	36.2	21.0	12.2	7.01	3.4	1.7	1.1				
1996–2000		91.5	95.6	70.2	51.7	32.4	19.7	11.4	6.5	3.4	1.6	0.8			
2001–2005			81.6	83.3	66.9	44.3	29.7	19.2	10.7	5.9	2.8	1.2	1.1		
2006–2010				77.7	77.9	52.1	38.9	27.4	16.1	9.9	5.4	2.8	1.1	0.5	
2011–2015					68.6	68.9	50.2	34.9	21.3	14.1	8.6	4.5	2.5	1.2	1.0
Females	Cohort														
Period	1913	1918	1923	1928	1933	1938	1943	1948	1953	1958	1963	1968	1973	1978	1983
1991–1995	47.3	41.3	28.5	17.0	12.4	7.6	4.6	2.6	2.0	1.3	1.1				
1996–2000		32.3	34.3	26.5	16.5	11.3	6.9	4.9	3.6	1.8	1.1	1.2			
2001–2005			33.5	30.3	20.5	14.0	10.2	6.8	4.4	3.0	2.2	1.1	0.8		
2006–2010				28.2	27.7	20.5	13.5	9.8	6.2	4.5	2.9	1.7	1.1	0.8	
2011–2015					23.1	26.7	16.7	13.3	9.4	6.1	4.8	2.6	1.8	1.2	1.2

### APC model by country

**Figure 1:**
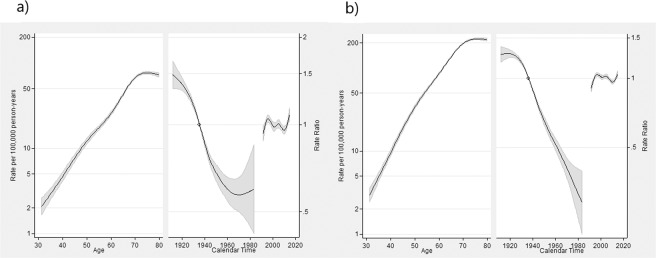
APC model fitting for gastric cancer data by sex (**a**: Females, **b**: Males) in Chile, 1990–2015 period. Age-related outcomes are expressed as specific-rates (from 30 to79 years old) per 100,000 person-years, and period of death, and cohort of birth effects interpreted as RR against the reference period (or reference cohort), which represents the median date of deaths between cases (or median date of birth among cases). For cohort effect, the reference categories were 1936.334 years for females (ASMR = 13.3) and 1935.334 for males (ASMR = 202.3), while 1995 and 2000 were those for the period effect in female and male populations, respectively. The leftmost solid line indicates the estimated age effect. Period of death and birth cohort effects are expressed in RR as solid lines (estimated effects, using reference indicated by a circle) on the right area of the graph, the longest of the solid lines show the estimated cohort effect, and the shortest line the estimated period effect. The regions adjacent to the lines provide the 95% confidence intervals.

Significant effects by sex were obtained for each country-APC analysis (p < 0.001). When considering females as a reference, estimates of MRR were 2.89 (95% CI 2.83; 2.94) and 2.62 (95% CI 2.58; 2.67) for Chile and Argentina, respectively. Age, cohort of birth, and period effects for Chile are shown in Fig. 1. APC models showed an estimated mortality rate ranging from less than 3.2 and 4.5 at age 30–39 for females and males, respectively, to about 116.6 and 276.7 at age 70 or older per 100,000 persons/years for females and males, respectively (Fig. 1). The age-effect curve showed a considerable increase in both sexes. Nonetheless, the notable overall decrease of birth-cohort effects patterns, in relation to the reference, were different between sexes, showing that younger cohorts of women stave off that risk reduction. Similar period effects were estimated for both sexes, revealing increasing RR in the first part of the study period (before 2000) and then decreasing afterward.

**Figure 2:**
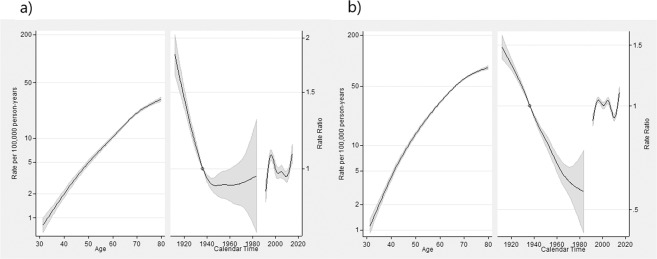
APC model fitting for gastric cancer data by sex (**a**: Females, **b**: Males) in Argentina, 1990–2015 period. Age-related outcomes are expressed as specific-rates (from 30 to79 years old) per 100,000 person-years, and period of death and cohort of birth effects interpreted as RR against the reference period (or reference cohort), which represents the median date of deaths between cases (or median date of birth among cases). Cohort references were 1938 and 1935 for females and males (ASMR equal to 7.61 and 68.5, respectively), also using the same reference (year 2000) for both sexes for period effect. The leftmost solid line indicates the estimated age effect, the longest of the solid lines on the RR half of the graph refers to the estimated cohort effect, and the shortest line in the RR half of the graph represents the estimated period effect. The circle indicates the reference point, and the regions adjacent to the lines provide the 95% confidence intervals.

For Argentina, different patterns for age, period, and cohort were observed for each sex (Fig. 2). The estimated mortality rate ranged from less than 2 to 5 at age 30–39 for females and males, respectively, to about 30.5 and 67.3 at age 70 or older per 100,000 person-years for females and males, respectively (Fig. 2). While rates for women were substantially lower than those for men, for all ages, this pattern was not as remarkable as it was in Chile. The effect of the birth cohort had not decreased since 1950 for females; moreover, it became stationary and showed the adjacent region of the MRR profile includes one. The cohort effect for males was similar to that of Chilean males, although less pronounced. When increasing RR in the first decade of study, females showed a different outline for period effect related to males, in both countries.

All models (by sex and country) attained lower AIC values when eight degrees of freedom were selected, suggesting a better fitting model.

### Joint APC model: country-effect assessment

**Figure 3:**
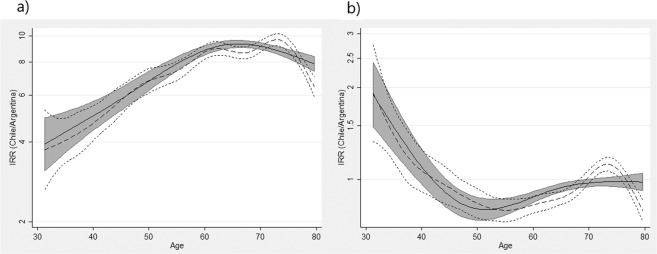
Graph of time-dependent MRR for the age-by-country interaction (**a**: Females, **b**: Males). Chile and Argentina, 1990–2015 period. The solid line is the MRR (Chile compared with Argentina) for the age-by-country interaction three degrees of freedom for splines (from the model using reduced splines for age and cohort, having fitted a model with eight degrees of freedom for each of the components). The shaded region indicates the 95% confidence interval. The dashed line gives the MRR by country in terms of the age component from the model with the full interaction by country using 8 degrees of freedom for each component. The dotted lines form the appropriate 95% confidence interval.

Finally, the APC model fitting performed for the complete dataset of mortality showed significant effects by country for both sexes (p < 0.001). Considering Argentina as the reference, MRR estimates were 7.08 (95%CI 6.95; 7.21) and 0.92 (95%CI 0.89; 0.94) for females and males, respectively. This result indicates that adjusted by time-components, GC mortality for females in Chile was noticeably higher than in Argentina, while an opposite effect was observed in males mainly for deaths occurring between 45 and 65 years old. Figure 3 illustrates time-dependent MRR for the age-by-country interaction and shows that the relationship between countries varies differently across age according to sex.

## Discussion

The present analysis focused on the age-standardized and sex-specific mortality rates for GC in two countries, Chile and Argentina, in the American Southern Cone. Overall, the highest rates (age-, cohort- or period-specific rates) were observed in Chile, where the rate values were at least two-fold higher than in Argentina. Mortality trends decreased in both sexes between 1990 and 2015 and more substantially in Chile and more favorably for males. Through the APC analysis, the present study confirmed the direct effect by age, with an increasing tendency in Chilean males; showed favorable decreasing mortality risk by cohorts in both countries; identifying that the younger female cohorts had a stabilized risk reduction since the 1950-cohort and similar increasing RR was found before 2000 and subsequent decreases for both countries. A main effect by country was obtained for both male and female populations. These findings confirm mortality differences between Chile and Argentina. GC represents 12.2% of all cancer deaths in Chile, while 4.7% in Argentina (2018)^5^. The number of cancer deaths is greatly influenced by the size and age composition of the population. In that sense, the APC model approach helps to understand the complex social, historical, and environmental factors that simultaneously impact individuals and populations^12^, integrating changes in age, calendar period, and birth cohort components.

Age and period effects have been sufficiently explored in cancer research. The results of the APC model are useful for regional comparison. In this situation, the different risk factors prevalence among birth cohorts should be taken into account^13^. Cohort effects reflect the long-established generational effect of risk factors in early life, which manifest as a progressive increase or decrease in cancer risk within the population. Our work provides several focuses to explore possible exposure sources.

In the present study, similar cohort patterns were observed in Chile as well as in Argentina, which indicates that shared exposure risk factors in early life may be responsible. Some risk factors have been recognized as main determinants of GC, such as *H. pylori* infection, smoking, body fatness, alcohol and salt consumption, and other specific dietary habits (consumption of fruit, processed meat, grilled or barbecued meat, fish, and foods preserved by salting)^8^.

*H. pylori* infection has been recognized as the primary risk factor for GC^14^. Higher proportions of cancer cases attributable to infections are found in South America (35.4% in 2012)^1^, and some evidence reports an overall decline in the prevalence of *H. pylori* infection in Latin America^15^. In particular, a decreasing trend in *H. pylori* prevalence has been attributed to changes leading to a higher socioeconomic status, better hygiene practices, and less household overcrowding^16^, as has been the case in Chile and Argentina in the last decades. Although there is no data on temporal trends of *H. pylori* infection for the whole population of these countries, a study performed in Buenos Aires (the most populated city in Argentina) showed a significant decrease in *H. pylori* rates from 2002 to 2009 in pediatric populations with gastrointestinal symptoms^17^. Also, Oporto *et al*.^18^ reported that in the Region de La Araucanía in Chile, one of the regions with the highest GC mortality rates in this country, *H. pylori* infection has decreased significantly, and Da Costa *et al*.^19^ observed a 16% reduction in prevalence in patients at the University of Chile’s Clinical Hospital over 11 years. Thus, the decrease observed in mortality trends (from cohort effect) could be explained, in part, by the reduction in *H. Pylori* prevalence.

Nonetheless, our findings reveal remarkably higher GC mortality rates by cohort group in Chile than in Argentina. It is noticeable that Chile still has a high prevalence of *H. pylori*, about 73% in adults^15^, like the prevalence found in some Asian countries^20^. In contrast, Argentina has around 36% in adults^21^. Besides, some studies have reported an increased risk of GC among populations who reside at high altitudes (as a surrogate for factors that might cluster in mountainous regions such as host genetics, bacterial, dietary, and environmental variables)^22,23^. Interestingly, the mean altitude of Santiago de Chile (the most populated city in Chile) is around 570 m above sea level, while for Buenos Aires (Argentina), the mean is 25 m.

Furthermore, differences observed in GC mortality rates (by age, period or cohort group) between Chile and Argentina could be related to the higher obesity prevalence in Chile (around 31.2% vs. 18% in Argentina, according to national surveys), a higher consumption of some foods with recognized promoting effects on GC occurrence (such as fish preserved by salting, broiled or charbroiled, and alcoholic beverages), and a lower citrus fruit intake (dietary protective factor for GC reported by the literature)^8^.

Beyond that, our findings show that Chile and Argentina follow a decreasing global tendency of GC mortality linked to the cohort effect; some differences between sexes should be highlighted. In Argentina according to the National Surveys of Risk Factors for chronic diseases, the prevalence of tobacco consumption in adults decreased from 29.7% in 2005 to 25.1% in 2013, being more prevalent in men (35.1% and 29.9% in 2005 and 2013, respectively) than in women (24.9% and 20.9%, respectively). This higher male prevalence in tobacco consumption was also highlighted in the 2012 Global Adult Tobacco Survey report in Chile. The stagnation of the cohort downward effect in women in both countries, a decade after it was reported in Europe^24^, maybe because women adopted smoking habits later compared to men and the decreasing age of onset of tobacco consumption in women^25^.

Period effects mostly reflect changes in the classification of diseases and advancements in diagnosis techniques or treatment^26^. Overall, the patterns of period rate ratio appear to be similar in Chile and Argentina, with an upward trend until around 2000, decreasing thereafter, which might be attributable to improvements in treatments and screening services. It has been suggested that GC survival rates are higher when there are early detection and diagnosis^8^. In Chile, some authors highlight the experience gained during the last decade by medical teams specialized in GC both for diagnosis and management of post-surgery adverse events^27^. Given that GC is ranked as the first cancer death cause (ranked by ASMR)^5^ among males in Chile, higher priority in the public health agenda was given for this country. Currently, Chile has two population-based interventions for GC treatment and *H. Pylori* treatment for symptomatic patients that were implemented in 2006 and 2013, respectively^28^. This scenario, in terms of public health, maybe a factor that contributes to explaining the rapid higher decreasing trend in males in this country, compared with Argentina, where focused strategies against this cancer type have not been defined.

As GC is a long-latent period disease, aging is a major risk factor influencing trends; in fact, GC is frequent in adults over the age of 50^8^. Consistently, in the present work, GC mortality rates increased exponentially with age, both by sex and country. From the APC approach, age effects reflect the biological and social processes of aging.

To our knowledge, there are no comparative studies between countries using the APC approach for cancer mortality in South America. Our work is the first to explore time-component effects in a joined analysis which can identify country-effect. One of the strengths of the present work is the use of natural cubic splines to overcome the known-called identifiability problem from the classical APC approach and to represent age, period, and cohort effects into a generalized linear model approach. Natural cubic splines are favored over step-functions, because they are flexible, and reflect smooth changes over time, which allow a more biologically plausible way of modeling non-communicable disease data^29^. However, it is useful to consider some methodological issues for the interpretation of cohort effects. A weakness of this work is that the study periods of the two countries analyzed were not completely matched, although this only occurred at the beginning of the time period (time-series starting in 1990 for Chile and 1991 for Argentina). Besides, it should be highlighted that the APC model has a systematic tendency to favor cohort effects as they have higher weight and a larger number of parameters; furthermore, changes in trends in recent cohorts should be interpreted with caution since the earlier and more recent cohorts are based on smaller numbers of deaths^24^.

From an epidemiological viewpoint, a risk factor perspective is more closely related to incidence than mortality trends. However, due to the absence of national cancer registers and the acceptable quality of mortality statistics in both countries (civil registration coverage of cause-of-death above 90%)^30^, mortality rates seem to be the most reliable option in Latin American cancer epidemiology research. An additional limitation was that the GC analysis was performed without distinguishing subtypes (cardiac and non-cardiac) when specific etiological pathways are recognized for each one. Concluding, our findings showed an overall favorable decreasing trend for GC mortality risk in the 1990–2015 period for Argentina and Chile. However, we emphasize that GC remains a significant health problem in the Southern Cone region, especially for Chile, where the burden of mortality (measured by age-, cohort-, and period-specific rates) was noticeably high. Several underlying factors that could explain similarities and differences between countries were pointed out and discussed by using the APC perspective; nonetheless, the stagnation of the cohort effect in women should be further evaluated. Thus, increased efforts focused on the high-risk population are needed in Chile, including cost-effective population-based interventions, such as *H. pylori* and endoscopic screening, for GC prevention and treatment, respectively^31^. At the same time, in both countries, public health interventions aimed at reducing the prevalence of potentially modifiable risk factors (such as tobacco use, unhealthy dietary intake, and alcohol consumption) should be maintained and strengthened over time^32^, considering gender imbalance when these interventions are targeting GC burden reduction. Finally, we highlight that the present analytical approach may be a valuable tool to be replicated in other countries with no population-based cancer registries and acceptable mortality data.

## Data Availability

The datasets generated and analyzed during the current study are available from the corresponding author on reasonable request.
